# Cerebral MRI in a prospective cohort study on depression and atherosclerosis: the BiDirect sample, processing pipelines, and analysis tools

**DOI:** 10.1186/s41747-023-00415-z

**Published:** 2024-02-09

**Authors:** Niklas Wulms, Harald Kugel, Christian Cnyrim, Anja Tenberge, Wolfram Schwindt, Udo Dannlowski, Klaus Berger, Benedikt Sundermann, Heike Minnerup

**Affiliations:** 1 Institute of Epidemiology and Social Medicine, University of Münster, Münster, Germany; 2 Clinic of Radiology Radiology, University Hospital Muenster, Münster, Germany; 3 Department of Clinical Radiology, Klinikum Ibbenbueren, Ibbenbueren, Germany; 4 Institute for Translational Psychiatry, University of Münster, Münster, Germany; 5 Institute of Radiology and Neuroradiology, Evangelisches Krankenhaus, Medical Campus, University of Oldenburg, Oldenburg, Germany; 6 Research Center Neurosensory Science, University of Oldenburg, Oldenburg, Germany

**Keywords:** Longitudinal studies, Magnetic resonance imaging, Medical image processing, Population health, Standardization

## Abstract

**Background:**

The use of cerebral magnetic resonance imaging (MRI) in observational studies has increased exponentially in recent years, making it critical to provide details about the study sample, image processing, and extracted imaging markers to validate and replicate study results. This article reviews the cerebral MRI dataset from the now-completed BiDirect cohort study, as an update and extension of the feasibility report published after the first two examination time points.

**Methods:**

We report the sample and flow of participants spanning four study sessions and twelve years. In addition, we provide details on the acquisition protocol; the processing pipelines, including standardization and quality control methods; and the analytical tools used and markers available.

**Results:**

All data were collected from 2010 to 2021 at a single site in Münster, Germany, starting with a population of 2,257 participants at baseline in 3 different cohorts: a population-based cohort (*n* = 911 at baseline, 672 with MRI data), patients diagnosed with depression (*n* = 999, 736 with MRI data), and patients with manifest cardiovascular disease (*n* = 347, 52 with MRI data). During the study period, a total of 4,315 MRI sessions were performed, and over 535 participants underwent MRI at all 4 time points.

**Conclusions:**

Images were converted to Brain Imaging Data Structure (a standard for organizing and describing neuroimaging data) and analyzed using common tools, such as CAT12, FSL, Freesurfer, and BIANCA to extract imaging biomarkers. The BiDirect study comprises a thoroughly phenotyped study population with structural and functional MRI data.

**Relevance statement:**

The BiDirect Study includes a population-based sample and two patient-based samples whose MRI data can help answer numerous neuropsychiatric and cardiovascular research questions.

**Key points:**

• The BiDirect study included characterized patient- and population-based cohorts with MRI data.

• Data were standardized to Brain Imaging Data Structure and processed with commonly available software.

• MRI data and markers are available upon request.

**Graphical Abstract:**

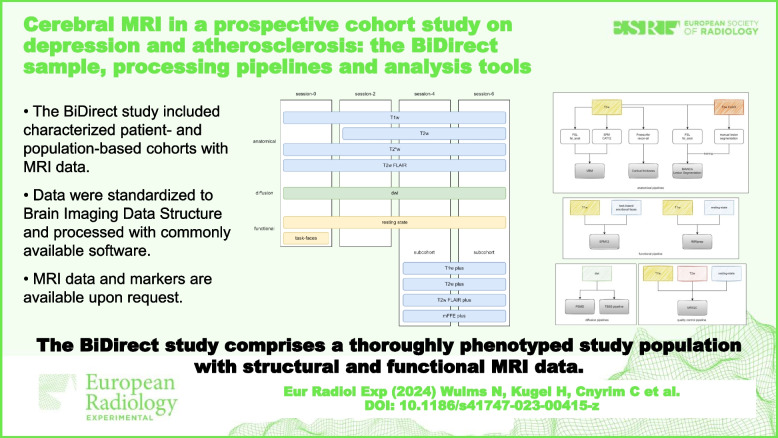

## Background

Transparent data description is important to promote reproducibility, replication, and collaboration in research. The present manuscript describes the sample, the acquisition protocols, the processing pipelines including quality control and standardization, and the applied analysis tools and derived markers of the MRI data of the now-completed population- and patient-based BiDirect cohort study, the latter first described in 2014 by Teismann et al. [1]. It is an update and extension of the feasibility report published after the first two examination time points and focused on rates and reasons of (non)participation in the MRI sessions [2]. The present manuscript complements this work by presenting the following: (1) the description of the last two of a total of four examination time points (follow-ups two and three) of the core MRI protocol; (2) the extended MRI data acquisition in a subsample (“plus” protocol) of follow-ups two and three; and (3) the final data handling and processing of the entire MRI data of the study. Another publication related to the descriptions presented here is an evaluation of the performance of the automated lesion segmentation algorithm (BIANCA) in our BiDirect MRI data by Wulms et al. 2022 [3].

The selection of an appropriate acquisition protocol depends on the specific research question and the imaging modality used. The STandards for ReportIng Vascular changes on nEuroimaging (STRIVE) recommend the use of T1-weighted (T1w), T2-weighted (T2w), and T2*-weighted (T2*w) sequences as well as fluid-attenuated inversion recovery (T2w FLAIR) and diffusion-weighted imaging (DWI) as minimally necessary sequences in large-scale epidemiological studies investigating small vessel disease and aging [4].

In addition to selecting an appropriate acquisition protocol, it is important to ensure that the protocol is executed consistently and that data quality is maintained over time. To achieve high-quality results, several quality control measures can be implemented throughout study acquisition, processing, and analysis. Image artifacts such as signal dropouts through motion [5] or tissue susceptibility variation [6] and scanner drift [7] can affect the quality of the data and should be identified and addressed manually or with automated tools such as MRIQC [8]. In addition, incidental findings should be identified and documented to allow flexible application of inclusion and exclusion criteria depending on the specific research question.

Another important aspect for reproducibility is the structure of the data [9]. Complex neuroimaging data offer many opportunities for structuring, processing, and analysis that compromise transparency and reproducibility. Therefore, in addition to a complete description of the acquisition protocol, the use of open software and frameworks is critical for reproducible neuroimaging [10, 11]. Adherence to a data organization standard, such as the Brain Imaging Data Structure (BIDS) specification [12], is also highly recommended for MRI data management.

The aim of this article is to provide comprehensive information on the MRI data from the monocentric prospective BiDirect study. This includes a detailed description of the sample over time, the imaging protocols, the data organization and quality control measures, and the analysis tools used and markers available.

## Methods

The study was approved by the Ethics Committee of the University of Münster and the Westphalian Chamber of Physicians in Münster, Germany. All participants gave written informed consent.

### Sample description

**Fig. 1:**
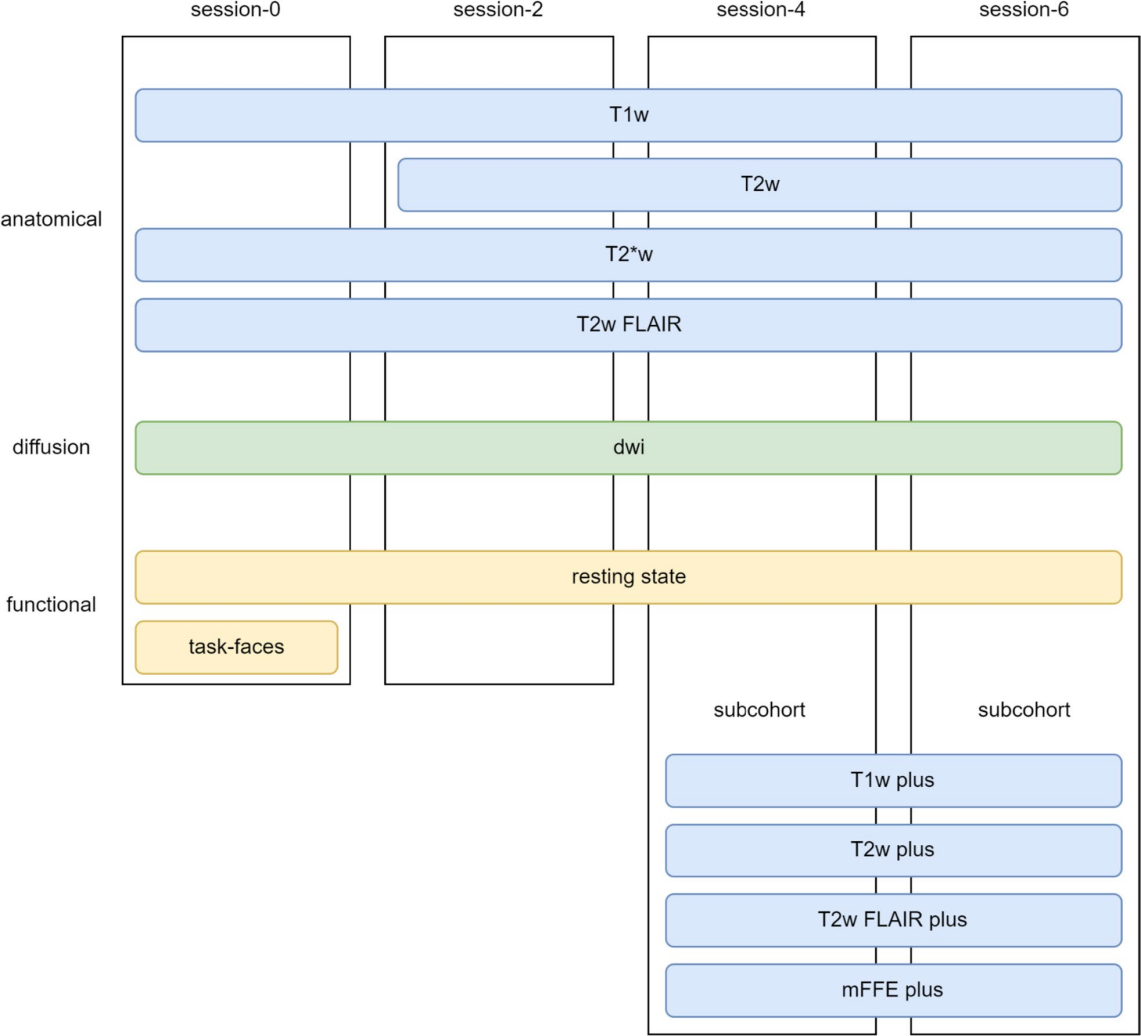
MRI sequences recorded during the four examination sessions. The different protocols are arranged from top to bottom, while the sessions are represented by four columns. The plus cohort was carried out only in sessions 4 and 6 within subcohorts from the population and depression cohorts

**Fig. 2:**
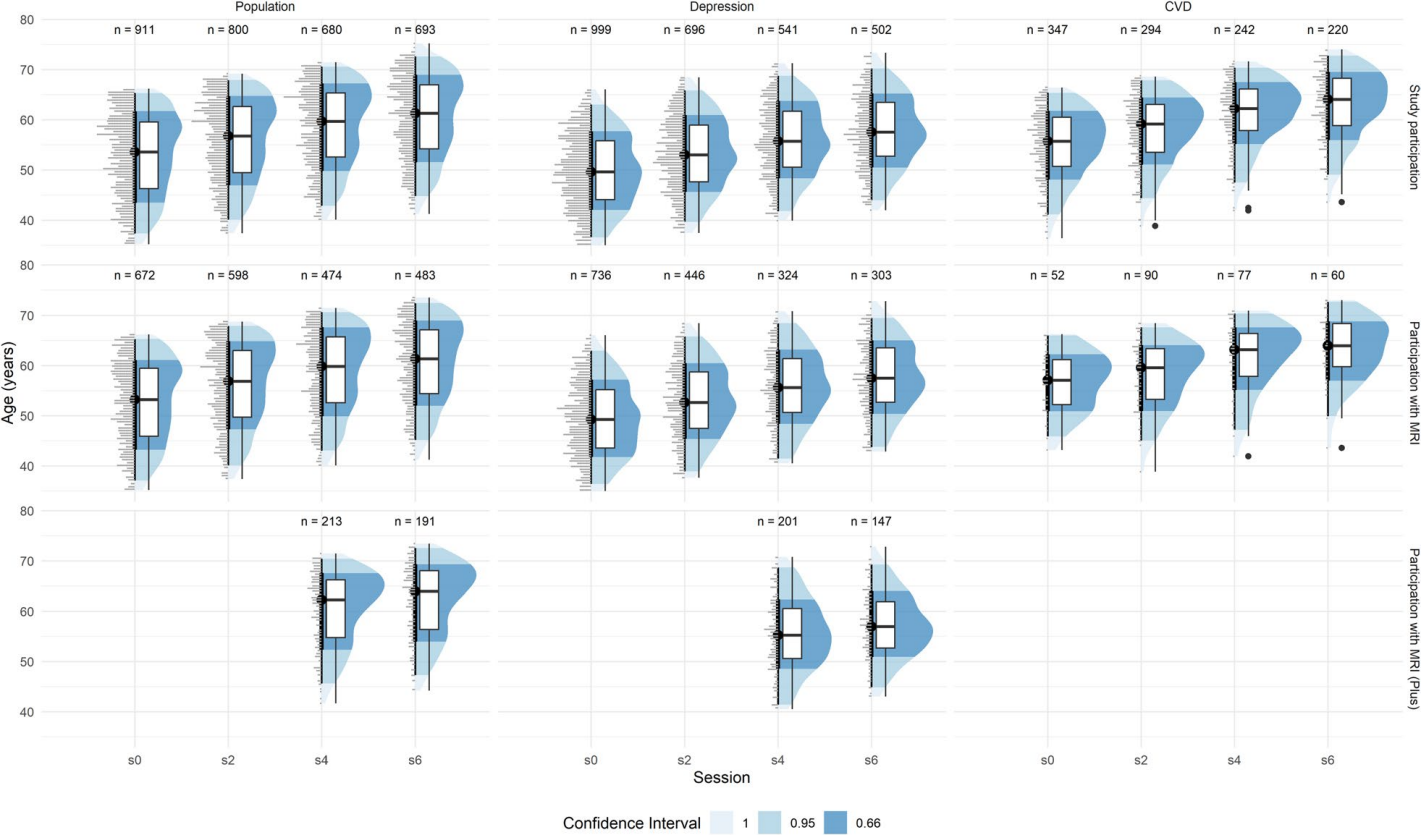
Distribution of age**-**stratified by session on the *x*-axis, cohort on the horizontal subplots, and available data on the vertical subplots (“all”—all BiDirect participants; “with MRI”—subset of BiDirect participants with MRI data; “with MRI Plus”—subset of BiDirect participants with MRI plus protocol data. Shown are boxplots with the median at each session and dotplots with a bin width of 0.5 years on the left. The color intensity of the distributions shows a confidence interval from 66 to 95%

The BiDirect study is a twelve-year monocentric prospective cohort study established to investigate the bidirectional association between subclinical atherosclerosis and depression. Starting in 2010, a cohort of residents (*n* = 911 at baseline) was randomly drawn from the population of Münster. A second cohort of participants with diagnosed depression (*n* = 999 at baseline) was recruited from psychiatric hospitals and outpatient services in and around Münster. A third cohort of patients with recently diagnosed acute cardiovascular disease was recruited from hospitals and rehabilitation facilities in and around Münster (*n* = 347 at baseline) (Table 1). A variety of examinations [1] were performed, including clinical, psychometric, and socioeconomic assessments as well as magnetic resonance imaging (MRI) of the brain [2]. All data were collected in four study sessions between 2010 and 2021 (Fig. 1). At baseline, participants were between 35 and 65 years of age (Fig. 2).

**Table 1:** Descriptive statistics of the BiDirect study presented stratified by acquisition time point (s0, s2, s4, s6). For each cohort and time point, the table includes information on the number of individuals in each cohort, sex, and age distribution

Session	Population				Depression				CVD			
	so	s2	S4	s6	so	s2	S4	s6	so	s2	S4	s6
BiDirect study population^a^	911 (100%)	800 (88%)	680 (75%)	693 (76%)	999 (100%)	696 (70%)	541 (54%)	502 (50%)	347 (100%)	294 (85%)	242 (70%)	220 (63%)
Sex^b^												
*Male*	448 (49%)	384 (48%)	329 (48%)	332 (48%)	406 (41%)	280 40%)	219 (40%)	205 (41%)	298 (86%)	252 (86%)	207 (86%)	186 (85%)
*Female*	463 (51%)	416 (52%)	351 (52%)	361 (52%)	593 (59%)	416 60%)	322 (60%)	297 (59%)	49 (14%)	42 (14%)	35 (14%)	34 (15%)
Age^c^ (years)	52.8 ± 8.2	55.9 ± 8.1	58.6 ± 8.0	60.4 ± 8.1	49.8 ± 7.3	53.2 ± 7.3	55.9 ± 7.3	57.8 ± 7.1	55.1 ± 6.7	58.1 ± 6.6	61.3 ± 6.3	63.0 ± 6.6
*Male*	52.6 ± 8.2	55.6 ± 8.2	58.3 ± 8.1	60.2 ± 8.0	49.5 ± 7.4	53.3 ± 7.3	56.5 ± 7.5	58.1 ± 7.2	55.0 ± 6.7	57.9 ± 6.6	61.2 ± 6.3	62.8 ± 6.6
*Female*	53.0 ± 8.2	56.1 ± 8.1	59.0 ± 8.0	60.6 ± 8.1	50.0 ± 7.2	53.1 ± 7.3	55.6 ± 7.2	57.5 ± 7.1	55.4 ± 6.7	58.8 ± 6.3	61.8 ± 6.7	63.9 ± 6.4
With MRI^a^	672 (100%)	598 (89%)	474 (71%)	483 (72%)	736 (100%)	446 (61%)	324 (44%)	303 (41%)	52 (100%)	90 (173%)	77 (148%)	60 (115%)
Sex^b^												
*Male*	322 (48%)	275 (46%)	235 (50%)	230 (48%)	301 (41%)	181 (41%)	131 (40%)	119 (39%)	41 (79%)	72 (80%)	65 (84%)	49 (82%)
*Female*	350 (52%)	323 (54%)	239 (50%)	253 (52%)	435 (59%)	265 (59%)	193 (60%)	184 (61%)	11 (21%)	18 (20%)	12 (16%)	11 (18%)
Age^c^ (years)	52.6 ± 8.2	56.0 ± 8.2	58.8 ± 8.1	60.5 ± 8.0	49.4 ± 7.3	52.9 ± 7.3	55.7 ± 7.2	57.6 ± 7.2	56.8 ± 5.9	58.1 ± 6.7	61.6 ± 6.8	63.3 ± 6.3
*Male*	52.2 ± 8.3	55.7 ± 8.2	58.2 ± 8.1	60.2 ± 8.0	48.8 ± 7.5	52.8 ± 7.4	56.2 ± 7.2	58.1 ± 7.0	56.8 ± 6.2	58.0 ± 6.9	61.3 ± 7.0	63.0 ± 6.7
*Female*	53.0 ± 8.1	56.3 ± 8.2	59.4 ± 8.1	60.8 ± 8.0	49.8 ± 7.1	53.0 ± 7.3	55.4 ± 7.3	57.4 ± 7.3	56.8 ± 4.7	58.8 ± 5.7	63.0 ± 5.8	64.6 ± 4.7
With MRI plus^a^			213 (100%)	191 (90%)			201 (100%)	147 (73%)				
Sex^b^												
*Male*			100 (47%)	87 (46%)			80 (40%)	65 (44%)				
*Female*			113 (53%)	104 (54%)			121 (60%)	82 (56%)				
Age^c^ (years)			60.4 ± 7.2	62.1 ± 7.3			55.4 ± 7.2	57.1 ± 6.5				
*Male*			59.4 ± 7.2	61.2 ± 7.3			56.2 ± 7.2	58.2 ± 6.7				
*Female*			61.2 ± 7.0	62.9 ± 7.2			54.8 ± 7.1	56.3 ± 6.2				

^a^ *n* (% of participation at baseline)

^b^ *n* (% at session, cross-sectional proportion)

^c^Mean ± standard deviation

### Acquisition

**Fig. 3:**
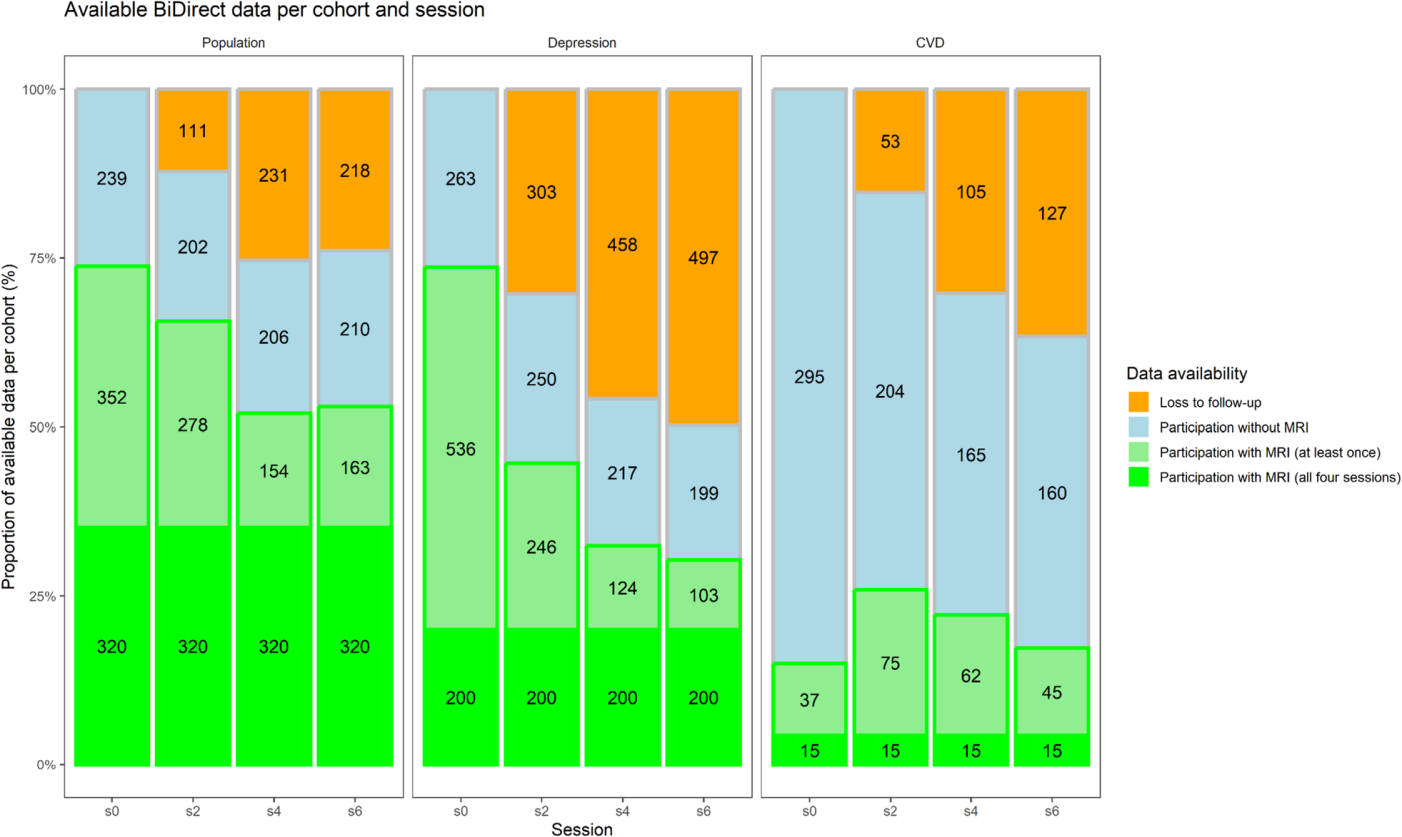
Bar chart of available MRI data stratified by session on the *x*-axis and cohort by horizontal subplot. The bars show the proportion of available or missing data per cohort and session from Table 1 on the *y*-axis. The numbers show the numbers of observations from each category. The *data availability* coloring of the bars shows loss to follow-up (orange), study participation without acquisition of MRI data (blue), and available MRI data (current session, light green; all four sessions, green)

**Fig. 4:**
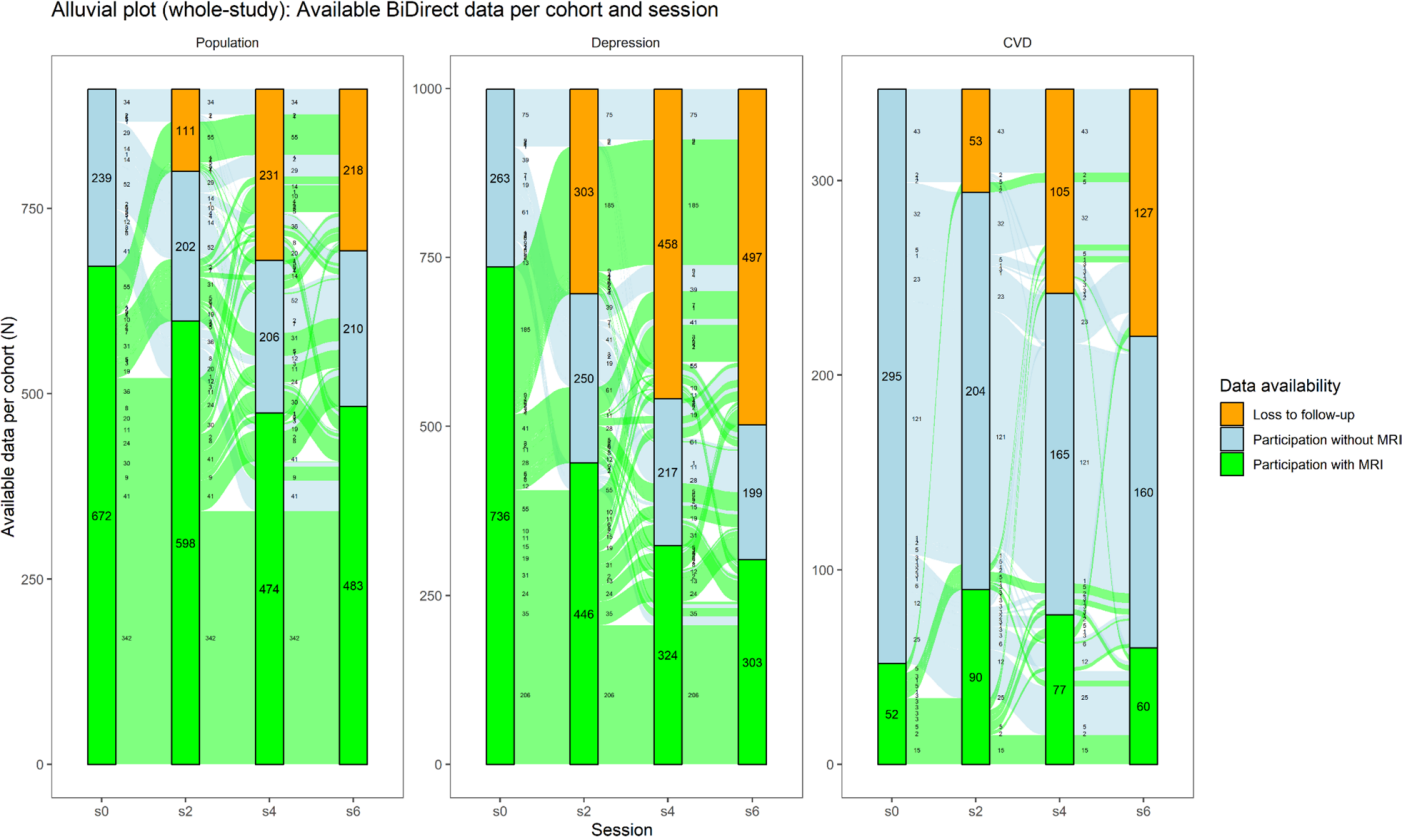
Alluvial plot of available data stratified by session on the *x*-axis and cohort by horizontal subplot. The bars (strata) show the proportion of available or missing data per cohort and session from Table 1 on the *y*-axis. The numbers in the strata show the observations in each category. The alluvia are lines that extend from s0 to s6 and contain the number of observations that fall into each category. The *data availability* coloring of the bars shows loss to follow-up (orange), study participation without acquisition of MRI data (blue), and available MRI data (green)

MRI of the brain was performed at each of the four examination sessions on the same 3T scanner (Philips Intera with Achieva upgrade, versions 2.5.3, 2.6.3, and 3.2.3) throughout the entire period at the University Department of Radiology, Münster University Hospital. The first feasibility report on the BiDirect MRI protocol was published in 2017 [2], focusing on sequence parameters and the study population in the first two examination sessions. For a detailed overview of the available data per sequence, cohort, and session, see Figs. 3 and 4 and Table 3.

### Core MRI protocol

As also previously described in a first feasibility report [2] 3D T1w, T2w FLAIR (T2w sequence with complete cerebrospinal fluid suppression), 2D T2*w, and 2D T2w sequences were used for anatomical imaging. In addition, a DWI sequence and a resting-state functional sequence (72 images at baseline and first follow-up and an extended version with 133 images at third and fourth follow-up) were performed. The parameters of the core protocol are listed in Table 2. All data were acquired with a single channel transmit/receive birdcage head coil.

**Table 2:** MRI acquisition parameters of the core protocol. The table is a licensed copy of supplementary Table S1 of [2], distributed under copyright, and redistributed with Springer Nature License 5,624,870,209,763

Sequence	Parameters				In-plane resolution			Slices			
	TR (ms)	TE (ms)	Tl (ms)	FA	Matrix	FOV (mm × mm)	Reconstructed (mm × mm)	*n*	Thickness (mm)	Gap (mm)	Orientation
2D T_2_^*^w gradient-echo (FFE)	574	16	-	18°	256 × 164	230 × 183	0.45 × 0.45	27	4	1	Axial
2D fast dark fluid imaging (TSE-FLAIR)	11,000	120	2,600	90°	352 × 206	230 × 186	0.45 × 0.45	27	4	1	Axial
2D T_2_w fast spin-echo (TSE)^a^	3,000	80	-	90°	400 × 255	230 × 184	0.45 × 0.45	27	4	1	Axial
3D T_1_w gradient-echo sequence with inversion prepulse (3D TFE)	7.26	3.56	404	9°	256 × 256	256 × 256	1.00 × 1.00	160	^b)^ 2	-	Sagittal
2D diffusion-weighted sequence with echo-planar imaging (single-shot SE-EPI)^c^	5,900	95	-	90°	128 × 128	240 × 240	0.94 × 0.94	36	3.6	-	Axial
Stimulation-based fMRI sequence with echo planar imaging (single-shot FFE-EPI), 82 volumes after 5 dummy scans^d^	2,200	30	-	90°	64 × 64	230 × 230	3.60 × 3.60	35	3.6	-	Axial
Resting-state fMRI sequence with echo planar imaging (single-shot FFE-EPI), 72 volumes after 5 dummy scans^e^	3,000	38	-	90°	64 × 64	230 × 230	3.60 × 3.60	36	3.6	-	Axial

*TR* Repetition time, *TE* Echo time, *TI* Inversion time, *FA* Flip angle, *FOV* Field of view, *fMRI* Functional magnetic resonance imaging

^a^1st follow-up examination and beyond

^b^Reconstructed by zero filling in k-space to 1-mm slice thickness

^c^20 gradient directions with *b* value of 1,000 s/mm^2^; reference *b* value is 0 s/mm^2^

^d^Baseline examination only. The emotion processing task is a short version of a previously published fMRI paradigm investigating neural responsiveness to happy and sad facial expressions in patients with major depression [1–3]. Facial stimuli consist of happy, sad, and neutral expressions according to Ekman and Friesen [4]. The passive viewing task with a presentation time of 3 min is subdivided into 6 blocks of 30 s each. During the first 20 s of a block, facial stimuli falling in the same category are presented for 500 ms each in a random sequence. The remaining 10 s of a block serve as no-face epoch. The order of blocks is sad-neutral-happy-sad-neutral-happy

^e^Prior to this sequence participants were instructed to remain motionless, mainly keep their eyes open, not to fall asleep, and not to think of anything in particular

### Emotion processing task

The emotion processing task was performed only at baseline (s0). It was a short version of a previously published functional MRI (fMRI) paradigm investigating neural responsiveness to happy and sad facial expressions in major depression [13, 14]. Facial stimuli consisted of sad, happy, and neutral expressions [14]. Subjects were presented with alternating 20-s epochs of a facial emotion category interleaved with 10-s epochs of a no-face baseline (crosshair). In a passive viewing task, facial stimuli were presented twice per second for 500 ms in a random sequence within each face category. Each 20-s face category epoch was followed by a 10-s no-face epoch and was presented twice, resulting in a total presentation time of 3 min. The order of blocks was sad-neutral-happy-sad-neutral-happy for each participant. For the emotion processing task, T2* functional data were acquired using a single-shot echo-planar sequence, with parameters selected to minimize distortion in the region of central interest, while retaining adequate signal-to-noise ratio and T2* sensitivity. Volumes consisting of 35 slices were acquired (parameters are listed in Table 2).

### MRI plus protocol

For approximately 200 randomly selected participants in each of the population-based and depression cohorts, an additional MRI protocol (BiDirect Plus, Table 4) with higher-resolution anatomic sequences including 3D T1w, 3D T2w, 3D FLAIR, and 3D (combined) multiecho fast field-echo was performed at follow-ups 2 and 3. Data were acquired using a six-channel phased array head coil. The plus protocol parameters are listed in Table 4.

### Quality control

There was no hardware upgrade after the start of the MRI study. The software updates did not alter the imaging features of the sequences reported here. A routine checkup of the scanner performance consisted of a mainly weekly Periodic Imaging Quality Test (PIQT) applying a vendor-provided head phantom measured in a birdcage head coil. The parameters tested were signal-to-noise ratio, geometric distortion, and floodfield homogeneity. The vendor service was called if the parameters exceeded specific limits defined by the vendor. During the lifetime of the scanner, the highest diagnostic image quality was maintained. Measures of quality control on manual segmentations of white matter hyperintensities (WMH) have been published in [3].

All images were reviewed for incidental findings by (neuro)radiologists in a setting comparable to routine clinical diagnostics. The description of this procedure and the respective results have been previously published in Teuber et al. [2]. An experienced team of neuroradiologists, neurologists, and epidemiologists met regularly to decide by consensus which findings were clinically relevant and should be reported to the participants [2]. The presence and nature of all incidental findings were also included in the study database to allow the adaptable application of inclusion and exclusion criteria for all subsequent data analyses.

All metadata were extracted from the DICOM headers and matched to the BiDirect database to avoid misclassification by ID, age, or sex and to check for deviations from standard MRI protocol (*e.g.*, different resolution, echo time, voxel size). We processed each image using the fully automated MRIQC pipeline [8] to assess image quality. In addition, we use the “BIDSconvertR” Shiny app [15] to provide quick visual access to each sequence and participant.

### Standardization

All MRI data was saved and synchronized weekly in DICOM format. The data was then converted to Neuroimaging Informatics Technology Initiative and structured into the BIDS specification [12] using the in-house developed R-package BIDSconvertR [15]. DICOM images were converted with dcm2niix (Linux; v1.0.20190902 [16]), and all potentially identifying information was removed from the header. All sequences were renamed and copied to the BIDS specification [12] and irrelevant sequences (*e.g.*, localizer) were discarded.

### Analysis tools and available markers

**Fig. 5:**
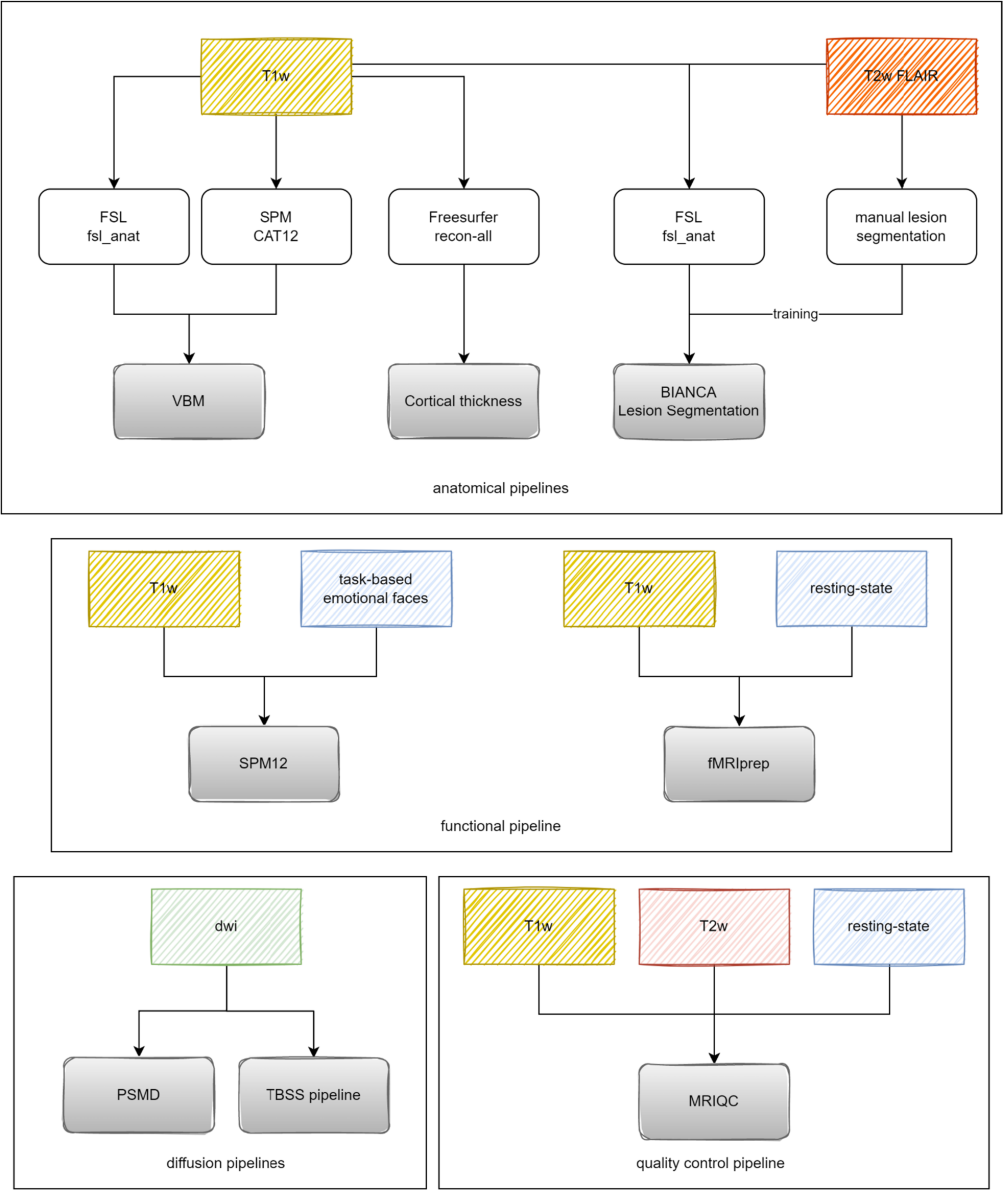
Neuroimaging pipelines: input sequence types, frameworks, and functions used

Structural and functional markers were derived only from the core protocol using established tools and pipelines (Fig. 5, Table 5).

### Anatomical pipeline

T1w data were processed with CAT12 for voxel-based morphometry [17] in developer mode to allow optional WMHs output. The “fsl_anat pipeline” of FSL (v6.0.3) [18–20] was used to process defaced T1w and T2w FLAIR images, which were then used to segment WMH in BIANCA [3, 21]. The fsl_anat-derived bias-corrected T1w images and the native T2w FLAIR/T2w/T2star images were extracted from the brain using “fsl_deface.” The T2w-derived brain masks were aligned to T1w space, and the transformation matrix was inverted and applied to the distorted T1w images to bring them into T2w space. The T1w images were downsampled to T2w space to be used with the T2w FLAIR images (required for BIANCA) for WMH segmentation. All 2D T2-weighted sequences (T2w, T2*w, T2w FLAIR) have the same resolution and voxel size. The brain mask was then realigned using the transformation matrix and applied to the bias-corrected T2-weighted sequences. Cortical thickness was calculated using Freesurfer (release v6.0 and v7.1.0, http://surfer.nmr.mgh.harvard.edu/). A user-defined function was used to extract all variables from the whole brain and specific regions of interest (ROIs) using different atlases.

### WMH segmentation

Two raters manually segmented WMHs in 201 T2w FLAIR images from the population-based cohort. These gold standard lesion segmentations were used to evaluate the performance of the automated lesion segmentation algorithm (BIANCA) as previously described by Wulms et al. [3]. We decided to use BIANCA after comparing the robustness of various white matter segmentation tools with respect to lesion volume estimation, which can be read here [22]. FSL BIANCA [21] was then trained with brain-extracted bias-corrected fsl_anat images (T1w, T2w FLAIR, same space, manual masks) based on the manually segmented lesion masks. The trained model was then applied to all other T1w and T2w FLAIR images (also bias-corrected, brain-extracted, in T2w FLAIR space) in the data set. The total lesion volume and lesion number were extracted from each image.

### Diffusion-weighted imaging

DWI data were processed with PSMD marker (v1.5) [23] to calculate the peak width of skeletonized mean diffusivity (PSMD) and mean skeletonized mean diffusivity (MSMD) values. The PSMD value and MSMD value were extracted, and the temporary file output argument was used to extract native and normalized fractional anisotropy (FA) and mean diffusivity (MD) images, as well as the skeletonized FA and MP maps for TBSS (tract-based spatial statistics). The normalized images were then used to extract mean FA and MD from the whole brain, white matter masks, TBSS images, and four ROI masks (MNI152 atlas: frontal, parietal, temporal, and occipital).

### Functional imaging pipeline

For the emotion processing task, a standard processing pipeline in SPM12 (https://www.fil.ion.ucl.ac.uk/spm) was implemented. Functional imaging data were motion-corrected, spatially normalized to standard MNI (Montreal Neurological Institute) space, and smoothed (Gaussian kernel, 8-mm FWHM (full width at half maximum)). For each subject, trials were averaged for each emotion condition. Brain responses to the emotion stimulus categories were isolated by convolving a vector of onset times of the sad, happy, neutral, and no-face conditions with a canonical hemodynamic response function. Two individual 1st level contrast images (happy-neutral, sad-neutral) were generated for 2nd level group statistics. Resting-state sequences were pre- and post-processed using fMRIPrep [24] using standard settings that deviate from the protocol only by turning off Freesurfer processing and usage (for detailed information, see supplementary material: fMRIprep boilerplate). Alternative postprocessing was performed in specific data analysis projects [25].

### Software and hardware

Ubuntu 18.04 LTS was used as the operating system. We also used locally installed versions of MATLAB (R2018b, The MathWorks, Inc., Natick, MA, USA), with SPM12 [26] including the CAT12 toolbox (r1742) [17], FSL (v6.0.3) [18–20], and Freesurfer (v6.0 and v7.1.0). We used pipelines for quality control and functional preprocessing in Dockerized versions: MRIQC (v0.16.0) [8] and fMRIPrep (v20.2.1) [24]. File management and FSL functions were wrapped and parallelized [27, 28] using the tidyverse library [29] in R (v4.2.1) [30]. CAT12 and PSMD computations were performed on a Dell Thinkstation-P520, Intel® Xeon(R) W-2125 (4.00GHz × 8 cores), 16 Gb DDR4-Ram. Freesurfer calculations were performed on a Dell Thinkstation-P500, Intel® Xeon(R) CPU E5-1650 v3 (3.50GHz × 12 cores), 16 Gb DDR4-Ram.

## Results

### Sample description

The distribution of age per cohort and study population is shown in Fig. 2. With all 4 study waves, BiDirect comprises a total of 6895 study examinations (49% women) with 4,315 MRI core protocols (53%) and 752 MRI plus protocols (56% women) (Figs. 3 and 4, Tables 1 and 3). In total, *n* = 320 of the population cohort and *n* = 200 of the depression cohort participated in all 4 MRI sessions of the core protocol (Fig. 3, Table 1).

**Table 3:** Descriptive statistics of available MRI data. The table shows the available data (expressed as a percentage of the total study population) by cohort and acquisition time point (s0, s2, s4, s6)

Session	Population				Depression				Cardiovascular disease			
	s0	s2	s4	s6	s0	s2	s4	s6	s0	s2	s4	s6
Total sample (*n*)	911	800	680	693	999	696	541	502	347	294	242	220
MRI data available^a^	672 (74%)	598 (75%)	474 (70%)	483 (70%)	736 (74%)	446 (64%)	324 (60%)	303 (60%)	52 (15%)	90 (31%)	77 (32%)	60 (27%)
T1w	672	596	474	483	736	442	323	302	52	89	77	60
T2w	5	597	474	483	4	444	324	302	-	89	77	60
T2*w	672	597	474	483	736	446	324	303	52	90	77	60
FLAIR	672	597	474	483	734	445	324	303	52	90	77	60
DWI	669	596	462	482	732	442	321	300	52	88	76	60
task-faces_BOLD	538	-	-	-	632	-	-	-	43	-	-	-
resting-state_BOLD	663	596	471	476	726	441	320	300	51	88	77	60
T1w_plus	-	-	212	191	-	-	201	147	-	-	-	-
T2w_plus	-	-	212	190	-	-	201	147	-	-	-	-
FLAIR_plus	-	-	213	191	-	-	201	147	-	-	-	-
mFFE_plus	-	-	213	190	-	-	201	143	-	-	-	-

^a^ *n* (% at session, cross-sectional proportion)

Due to termination through participants, technical reasons, motion artifacts, or altered parameters, some sequences were missing or discarded. Further information on contraindications and other reasons for nonparticipation in MRI examinations are listed in the MRI feasibility report of the BiDirect study [2]. During the 12 years of follow-up, starting from a study population of 2,257 participants, 842 participants (37%) were lost resulting in 1,415 participants at the last follow-up (Fig. 3). Regarding MRI data, 1,460 MRI sequences were acquired at baseline and 846 MRI sequences were acquired at the last follow-up, resulting in 614 participants (42%) lost to follow-up.

The population cohort with MRI lost 74 participants (11%) between baseline (s0) and first follow-up (s2), 198 participants (29%) between baseline (s0) and second follow-up (s4), and 189 participants (28%) between baseline and third follow-up (s6). The depression cohort with MRI lost 290 participants (39%) between baseline (s0) and first follow-up (s2), 412 participants (56%) between baseline (s0) and second follow-up (s4), and 433 participants (59%) between baseline and third follow-up (s6). The CVD cohort with MRI gained 38 participants (73%) between baseline (s0) and first follow-up (s2), 25 participants (48%) between baseline (s0) and second follow-up (s4), and 8 participants (15%) between baseline and third follow-up (s6).

### Acquisition and processing

The parameters of the MRI core protocol are listed in Table 2 and have previously been published in the BiDirect MRI feasibility report by Teuber et al. [2]. The parameters of the MRI plus protocol are summarized in Table 4. Table 5 gives an overview of the frameworks, tools, and analysis pipelines used.

**Table 4:** MRI acquisition parameters of the PLUS protocol

Sequence	Parameters				In-plane resolution			Slices			
	TR (ms)	TE (ms)	Tl (ms)	FA	Matrix	FOV (mm × mm)	Reconstructed (mm × mm)	*n*	Thickness (mm)	Gap (mm)	Orientation
T1-weighted 3D TFE	7.6	3.5		9°	256 × 255	256 × 256	512 × 512	320	1	0	Sagittal
T2-weighted 2D	3,000	80		90°	292 × 190	240 × 200	512 × 426	75	2	0.2	Transverse
FLAIR 3D	8,000	332	2,400	Variable	228 × 226	250 × 250	576 × 576	300	0.6	0	Sagittal
3D multiecho FFE	54	5.2/11.6/18.0/24.4/30.8/37.2/43.6		20°	240 × 187	240 × 188	512 × 400	60	2	0	Transverse

*FA* Flip angle, *FFE* Fast field echo, *FLAIR* Fluid-attenuated inversion recovery, *FOV* Field of view, *TE* Echo time, *TFE* Turbo field echo, *TI* Inversion time, *TR* Repetition time, *TSE* Turbo spin echo

**Table 5:** Neuroimaging pipelines: frameworks, tools, analysis types, output variables

Processing step	Framework	Tool	Input	Type	Variable/biomarker
Standardization: NII and BIDS conversion	R	BIDSconvertR [15]	dicom	dcm2niix [16] BIDS-conversion	json-metadata, id, birthdate, weight
Quality control	Docker	MRIQC [8]	T1w, T2w, bold	MRIQC pipeline	See [8]
Anatomical pipelines	SPM	CAT12 [14]	T1w	VBM	Volume (native/normalized): GM, WM, CSF, WMH + mask
	FSL	fsl_anat	T1w, T2w FLAIR	Anatomical pipeline	Volume (native/normalized): GM, WM, CSF
	Freesurfer (v6 and v7.1.0)	recon-all (surfer.nmr.mgh.harvard.edu/)	T1w	Cortical thickness	Cortical thickness, ROI-wise
Lesion delineation pipelines	SPM	CAT12 [17]	T1w	Lesion segmentation (intensity-based)	Volume (native/normalized): WMH + mask
	FSL	BIANCA [21]	T1w (BET, denoised, FLAIR space)	Lesion segmentation (trained—KNN)	Lesion count + volume (ml) + mask
			T2w FLAIR (BET, denoised)		
Diffusion-weighted pipelines	FSL	PSMD-Marker [23]	DWI + .bval + .bvec	Diffusion-weighted imaging	PSMD, MSMD, FA/MD (native/normalized and ROI-wise),
					TBSS
Functional pipelines	Docker	fMRIprep [24]	T1w + bold	Anatomical and functional preprocessing	Structural and functional derivatives; see [24]
				With (disabled Freesurfer processing)	

*BET* Brain-extracted, *CSF* Cerebrospinal fluid, *DWI* Diffusion-weighted image, *FA* Fractional anisotropy, *FLAIR* Fluid-attenuated inversion recovery, *Gm* Gray matter, *KNN* K-nearest neighbors, *MD* Mean diffusivity, *ROI* Region of interest, *T1w* T1-weighted-image, *T2w* T2-weighted image, *TBSS* Tract-based spatial statistics, *VBM* Voxel-based morphometry, *Wm* White matter

### Available markers

Extracted neuroimaging markers (Table 5) include both structural and functional markers, such as gray matter volume (CAT12), WMH volume (BIANCA), cortical thickness (Freesurfer), and measures of functional connectivity (fMRIprep). WMH lesion segmentation pipelines extracted measures of lesion volume, lesion count, and the actual three-dimensional lesion map. In addition, diffusion-weighted imaging pipelines extracted measures of microstructural integrity, including fractional anisotropy and mean diffusivity.

## Discussion

The BiDirect study features a unique combination of three cohorts of middle-aged men and women captured across four examinations over twelve years. Compared with other cohort studies using cerebral MRI, it is at the upper end of the sample size range [31] with a total number 6,895 imaging sequences from 1,460 subjects (672 from the general population, 736 with depression, and 52 with cardiovascular disease) with MRI data at baseline. The Human Connectome Project (HCP) collected data from 1,100 volunteer participants starting in 2010 [32]. The prospective Rotterdam Scan Study examined imaging markers from 5,286 population-based participants from the Ommoord neighborhood in Rotterdam. The German National Cohort recruited 205,000 participants at 18 study sites in Germany [33] via population registers. At baseline, 56,971 participants underwent in-depth phenotyping and 30,861 of them participated in 3-T MRI of the brain [34]. The German Rhineland Study also targets 30,000 subjects [35]. The UK Biobank collected data from about 500,000 volunteer participants and in 2014 began inviting 100,000 of those original volunteers for brain, heart, and body imaging [36]. Imaging data from 10,000 volunteers has already been processed and made available [36].

Regarding follow-up losses, the population cohort lost 218 participants (24%) between baseline (s0, *n* = 911) and last follow-up (s6, *n* = 693). Among participants with MRI, there was a loss of 189 participants (28%, s0 = 672 and s6 = 483). In comparison, the Rotterdam Scan Study showed a decrease from 3,932 participants (2,956 with MRI) to 3,122 participants (1,854 with MRI) over 10 years from 2005 to 2015 [37], corresponding to a loss to follow-up of 810 participants (21%) from the total cohort and of 1,102 participants with MRI (37%).

The cardiovascular disease cohort lost 127 participants (37%) between baseline (s0, *n* = 347) and last follow-up (s6, *n* = 220). However, they gained 8 participants with MRI (15%) between s0 (*n* = 52) and s6 (*n* = 60). This gain resulted from contraindications, such as newly implanted coronary stents, which made them temporarily unavailable for MRI [2]. However, all participants were given the opportunity to participate in an MRI session at a subsequent follow-up visit.

The depression cohort lost 497 participants (50%) between baseline (s0, *n* = 999) and last follow-up (s6, *n* = 502). Among participants with MRI, there was a loss of 189 participants (59%, s0 = 736 and s6 = 303). This 1.4- to 2.4-fold higher probability of dropout compared with the population-based cohort was expected because of the underlying disease [38].

In BiDirect, we used T1-weighted, T2-weighted, and diffusion-weighted sequences to measure anatomic features and white matter connectivity. Thus, the MRI protocol complies with STRIVE criteria [4]. We also acquired two functional sequences, a task-based paradigm with emotional faces (baseline only) and a resting-state sequence. WMH were extracted using BIANCA, a widely used and validated tool [3, 21]. During the study period, higher-resolution imaging techniques were increasingly used in routine clinical practice [39]. In follow-up visits 2 and 3, we therefore added high-resolution imaging sequences for a subcohort of approximately 400 participants.

All MRI data from the BiDirect study were standardized to BIDS using the BIDSconvertR [15]. The BIDS specification [12] is a widely used tool for organizing neuroimaging data that is being actively developed by the BIDS consortium. We applied easy-to-use, widely available, and open-access pipelines (*e.g.*, BIDS apps [39]) developed for or adapted to BIDS structured data to improve the reproducibility of our data.

The study is associated with certain limitations. The sequences used were not updated during the study period and were therefore increasingly outdated, except for the plus protocol. We did this intentionally to ensure optimal comparability over time. In addition, the T2w images were not acquired at baseline. Moreover, given the large number of images acquired, we did not perform manual quality control or image quality assessment. This is left to the individual scientist for each specific project.

The present manuscript also needs to be distinguished from previous work, mainly the MRI feasibility report by Teuber et al. [2], which presented the MRI data acquisition of the first two examination time points together with the rates and reasons of MRI non-participation, as well as the report on the evaluation of the performance of the automated lesion segmentation algorithm (BIANCA) in our MRI data by Wulms et al. [3].

The BiDirect study comprises a thoroughly phenotyped study population with structural and functional MRI data. The imaging data is standardized to the BIDS specification and already processed with the most common analysis tools. Both the images and the MRI markers are available for collaboration and sharing.

## Data Availability

The data are not publicly available due to GDPR regulations. However, the data can be made available for collaboration upon request from the Institute for Epidemiology and Social Medicine, University of Münster, Germany.

## Abbreviations

2D: Two-dimensional
3D: Three-dimensional
BIDS: Brain Imaging Data Structure
DICOM: Digital Imaging and Communications in Medicine
DWI: Diffusion-weighted imaging
FA: Fractional anisotropy
FLAIR: Fluid-attenuated inversion recovery
fMRI: Functional MRI
MD: Mean diffusivity
MRI: Magnetic resonance imaging
MSMD: Mean skeletonized mean diffusivity
PSMD: Peak width skeletonized mean diffusivity
ROI: Region of interest
STRIVE: STandards for ReportIng Vascular changes on nEuroimaging
T1w: T1-weighted
T2*w: T2*-weighted
T2w: T2-weighted
TBSS: Tract-based spatial statistics
WMH: White matter hyperintensities
